# A Protocol to Shorten Rice Growth Cycle in Plant Factories: An Integrated Study of Light, Planting Density and Phytohormone Regulation

**DOI:** 10.3390/plants15030343

**Published:** 2026-01-23

**Authors:** Gongzhen Fu, Pengtao Zheng, Feng Wang, Jinhua Li, Xing Huo, Yanxia Xiao, Yilong Liao, Manshan Zhu, Chongyun Fu, Xueqin Zeng, Xiaozhi Ma, Le Kong, Leiqing Chen, Xueru Hou, Wuge Liu, Dilin Liu

**Affiliations:** 1 College of Agriculture, South China Agricultural University, Guangzhou 510642, China; 13617766417@163.com (G.F.); 17774605480@163.com (P.Z.); xyx13759419730@163.com (Y.X.); 2 Rice Research Institute, Guangdong Academy of Agricultural Sciences, Guangzhou 510640, China; fwang1631@163.com (F.W.); lijinhua-2002@163.com (J.L.); huoxing@foxmail.com (X.H.); liaoyilong@21cn.com (Y.L.); zhumanshan@tom.com (M.Z.); fcysd2901@126.com (C.F.); sszxq@126.com (X.Z.); xiaozhima_1223@sina.com (X.M.); kongldk@163.com (L.K.); chenleiq0816@163.com (L.C.); hxr118123@163.com (X.H.); 3 Guangdong Key Laboratory of Rice Science and Technology, Guangzhou 510640, China; 4 Guangdong Rice Engineering Laboratory, Guangzhou 510640, China; 5 Key Laboratory of Genetics and Breeding of High Quality Rice in Southern China (Co-Construction by Ministry and Province), Guangzhou 510640, China; 6 College of Plant Science, Xizang Agricultural and Animal Husbandry University, Linzhi 860000, China

**Keywords:** breeding efficiency, LED, speed breeding, life cycle, plant factory

## Abstract

Speed breeding represents a pivotal technology for enhancing crop breeding efficiency. This study systematically examined the regulation of LED light environments, planting density, and gibberellic acid (GA_3_) on rice growth cycle progression in plant factories, establishing an integrated speed breeding protocol. The experimental design comprised three components: (1) coupling seedling age (9–25 days, variety-dependent) with LED environments and planting densities (25–100 plants/tray); (2) combining light intensity gradients (450 and 900 μmol·m^−2^·s^−1^) with photoperiod control; (3) applying GA_3_ gradients (0–120 ppm) to enhance immature seed germination. Results indicated that high planting densities (>50 plants/tray) prolonged the growth cycle and decreased yield, whereas 25 plants/tray optimally balanced growth cycle shortening and yield maximization. Under short-day induction, Nipponbare (Nip) and Wufeng B (WFB) reached heading at 39 and 58 days after sowing (DAS), respectively. Stage-specific light responses were observed: 450 μmol·m^−2^·s^−1^ during the basic vegetative phase (BVP) promoted morphological development, whereas 900 μmol·m^−2^·s^−1^ during the photoperiod-sensitive phase (PSP) accelerated tillering and panicle differentiation. GA_3_ treatment (60 ppm) enhanced the germination rate of immature seeds by 31%. The optimized lightregimes comprised natural light + 900 μmol·m^−2^·s^−1^ (NL–900) and 450 μmol·m^−2^·s^−1^ + 900 μmol·m^−2^·s^−1^ (450–900), combined with density control (25 plants/tray) and GA_3_-mediated immature seed utilization, shortened the generation time to 54 days and 70 days for Nip and WFB, respectively. This integrated protocol establishes an efficient strategy for rice speed breeding in plant factories.

## 1. Introduction

Rice is a staple food for over half of the global population. The world population is projected to reach 10 billion within 30 years, representing a 25% increase [1]. This growth drives demand for rice varieties with higher yield and improved quality. Conventional breeding faces efficiency limitations due to climatic fluctuations and extended breeding cycles (10–20 years). Speed breeding (SB) addresses these constraints by shortening growth cycles to enable multiple generations annually and has been validated in various crops [2]. LED plant factories provide precise control over both light and non-light environmental factors, offering an ideal SB platform [3]. Although LED lighting is extensively used in horticultural production, its application in crop breeding remains exploratory [4]. The growing demand for breeding efficiency and genetic gain positions LED-SB technology as a frontier approach capable of reshaping global crop breeding paradigms.

Extensive research underscores the profound impact of tailored environmental control on plant physiology and productivity. For example, purple light with far-red wavelengths significantly enhanced flowering in *Viola cornuta* ‘Penny Red Wing’, outperforming white light spectra [5]. In lettuce, optimizing light intensity and photoperiod increased biomass, improved morphology, and elevated nutritional quality. Optimal combinations, such as 290 μmol·m^−2^·s^−1^ with specific photoperiods, promoted superior growth [6,7]. For holy basil, 0.4% KNO_3_ priming improved germination and early growth [8]. In strawberry, stage-specific light optimization (90 μmol·m^−2^·s^−1^ for rooting; 270 μmol·m^−2^·s^−1^ for seedling growth) enhanced propagation efficiency [9]. These capabilities directly address the time constraints of conventional breeding. By decoupling growth from seasonal limitations, controlled environments provide an ideal platform for implementing Speed Breeding [10].

Speed breeding advancements have been documented in multiple crops, including rapeseed [11], wheat [12], cannabis [13], and rice [14]. Interactions between light recipes (spectrum, intensity, photoperiod) and non-light factors have been examined in rice, establishing a referential framework for speed breeding [15,16]. However, integrated optimization of multiple environmental factors in plant factories requires further investigation [4]. Comprehensive protocols integrating LED-based light regulation with non-light environmental controls are needed to accelerate growth and shorten the rice life cycle. Existing light recipes, for instance red/blue ratio of 2:1, 800 PPFD and 10 h short day, significantly shorten the rice growth cycle [15]. Far-red light supplementation further promotes heading in certain rice varieties [17]. Rice development progresses through vegetative, reproductive, and maturation phases. The vegetative phase comprises basic vegetative (BVP) and photoperiod-sensitive (PSP) phases. Seedlings are photoperiod-insensitive during BVP, but transition to PSP enables short-day-induced early flowering [15].

The present study addresses the following key issues: (1) Using plug-tray cultivation to systematically examine the effects of different light intensities across distinct rice growth stages, while simultaneously increasing planting density to expand the effective plant population size. (2) Unlike the fixed duration of BVP, given the genotypic variation in PSP, identifying the optimal seedling age for short-day induction is critical to balancing vegetative and reproductive growth and shortening the life cycle. (3) Early harvesting after heading—GA_3_ treatment of immature seeds markedly enhances germination rates. (4) Integrated strategies combining light environment parameters (spectrum, intensity, photoperiod) with non-light factors (temperature, humidity, hormones, planting density, nutrition). This study systematically validated the feasibility of high-density plug tray cultivation in plant factories, clarified the differential responses of indica and japonica rice to stage-specific light regulation, and, by integrating exogenous GA_3_ treatment to promote early seed harvest, established for the first time a “light–density–hormone” coordinated protocol for accelerating rice growth cycles under LED-based artificial lighting. This provides a reproducible and efficient technical approach for rice breeding.

## 2. Results

### 2.1. Effects of LED Light Intensity on Morphogenesis at Different Rice Growth Stages

**Figure 1:**
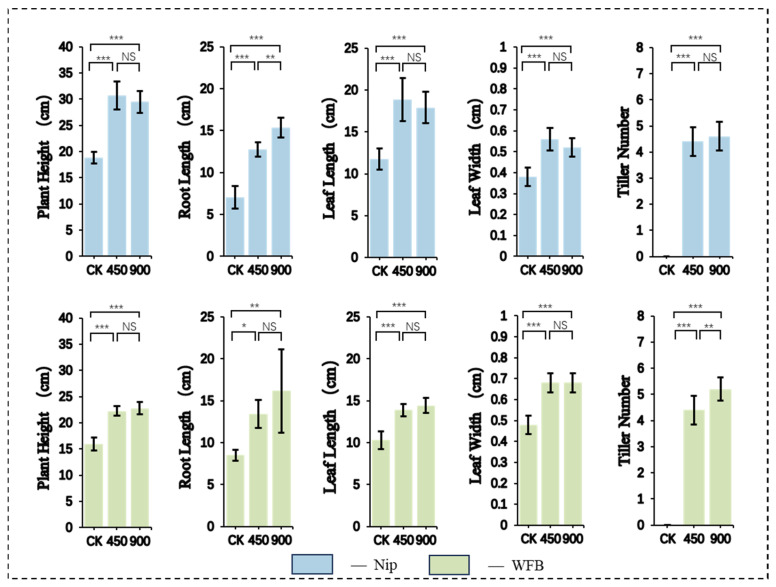
Effects of different light intensities on rice growth during the basic vegetative phase (BVP). Color codes represent distinct rice cultivars. CK: natural light control; 450 and 900: LED light intensities with units of μmol·m^−2^·s^−1^. NS: not significant (*p* ≥ 0.05). The symbols *, **, and *** indicate statistical significance at *p* < 0.05, *p* < 0.01, and *p* < 0.001, respectively.

During the basic vegetative phase (BVP), plants were maintained under 24 h long-day conditions in the LED–speed breeding (LED-SB) system (Figure 1). both Nip and WFB exhibited significantly greater plant height, root length, leaf length, leaf width, and tiller number under 450 and 900 μmol·m^−2^·s^−1^ PPFD compared to CK. The two cultivars responded differently to light intensity. For Nip, no significant differences were observed in plant height, leaf length, leaf width, or tiller number between 450 and 900 μmol·m^−2^·s^−1^, though the former showed higher values for plant height, leaf length, and leaf width, while the latter produced more tillers and significantly longer roots. For WFB, no significant differences were detected in plant height, root length, leaf length, or leaf width between the two light intensities, but 900 μmol·m^−2^·s^−1^ resulted in significantly more tillers than 450 μmol·m^−2^·s^−1^.

**Figure 2:**
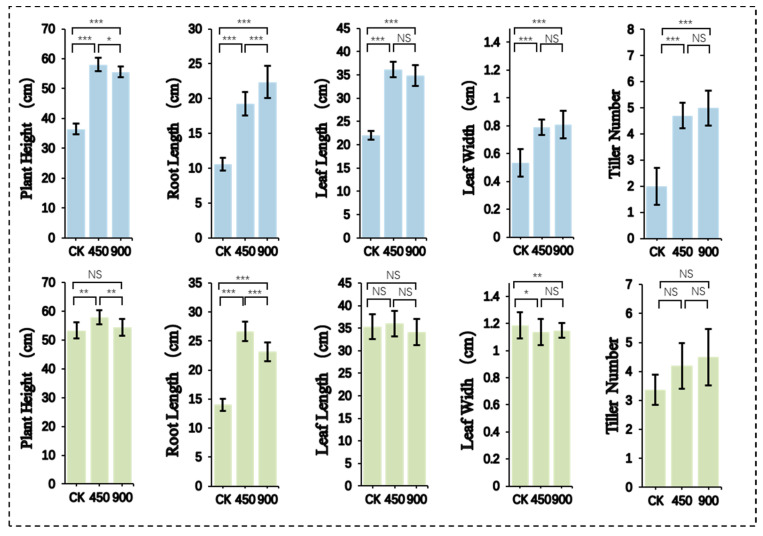
Effects of different light intensities on rice growth during the photoperiod-sensitive phase (PSP). Colors represent distinct rice cultivars. CK: natural light control; 450 and 900: LED light intensities with units of μmol·m^−2^·s^−1^. NS: not significant (*p* ≥ 0.05). The symbols *, **, and *** indicate statistical significance at *p* < 0.05, *p* < 0.01, and *p* < 0.001, respectively.

During the photoperiod-sensitive phase (PSP), plants were exposed to 10 h short-day conditions (Figure 2). Nip and WFB responded differently under CK, 450, and 900 μmol·m^−2^·s^−1^ conditions. For Nip, no significant differences in leaf length, leaf width, or tiller number were found between 450 and 900 μmol·m^−2^·s^−1^, though both were significantly higher than CK. The 450 μmol·m^−2^·s^−1^ treatment yielded the longest leaves, while 900 μmol·m^−2^·s^−1^ produced the most tillers. Plant height at 450 μmol·m^−2^·s^−1^ was significantly greater than CK and 900 μmol·m^−2^·s^−1^, whereas root length was highest under 900 μmol·m^−2^·s^−1^. For WFB, no significant differences in leaf length, leaf width, or tiller number were observed between 450 and 900 μmol·m^−2^·s^−1^, though 900 μmol·m^−2^·s^−1^ resulted in the most tillers and 450 μmol·m^−2^·s^−1^ the longest leaves. Plant height and root length at 450 μmol·m^−2^·s^−1^ were significantly greater than CK and 900 μmol·m^−2^·s^−1^.

### 2.2. Growth Responses of Rice to Different Planting Densities Under the LED-SB Platform

**Figure 3:**
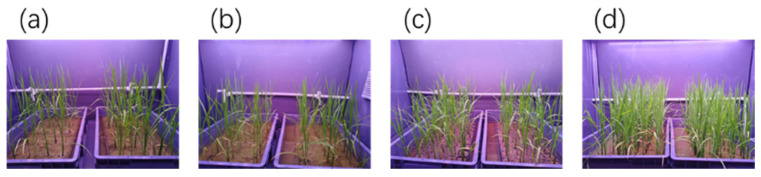
Seedling growth with different planting densities under LED lighting conditions. (**a**) 25 plants/tray (PD-25), with a planting area of 60.48 cm^2^ per plant; (**b**) 30 plants/tray (PD-30), with a planting area of 50.4 cm^2^ per plant; (**c**) 50 plants/tray (PD-50), with a planting area of 30.24 cm^2^ per plant; (**d**) 100 plants/tray (PD-100), with a planting area of 15.12 cm^2^ per plant.

To examine planting density effects on rice growth under LED-speed breeding (LED-SB) platform, four planting density gradients were established: PD-25, PD-30, PD-50, and PD-100 (specific density values per unit area are provided in Figure 3). Relative to the baseline density PD-25, PD-50 significantly reduced key agronomic traits (all *p* < 0.05; Table 1): plant height by 11.56%, panicle length by 4.5%, panicle exsertion by 45.1%, panicle number by 44.1%, and tiller number by 30.2%. A further increase in density to PD-100 led to more pronounced reductions in these parameters (all *p* < 0.01 vs. PD-25; Table 1), with decreases of 18.6%, 15.4%, 88.1%, 70.6%, and 69.8%, respectively, compared to PD-25.

Growth duration exhibited a density-dependent increasing trend (Table 1): PD-25 showed the shortest growth duration at 70.7 ± 2.58 d, while PD-30, PD-50, and PD-100 had longer durations of 81.44 ± 1.33 d, 77.89 ± 1.76 d, and 81.3 ± 1.89 d, respectively. Among these, PD-30 and PD-100 had significantly longer growth durations than PD-25 (*p* < 0.05), whereas the difference between PD-50 and PD-25 was not significant (NS, *p* ≥ 0.05).

**Figure 4:**
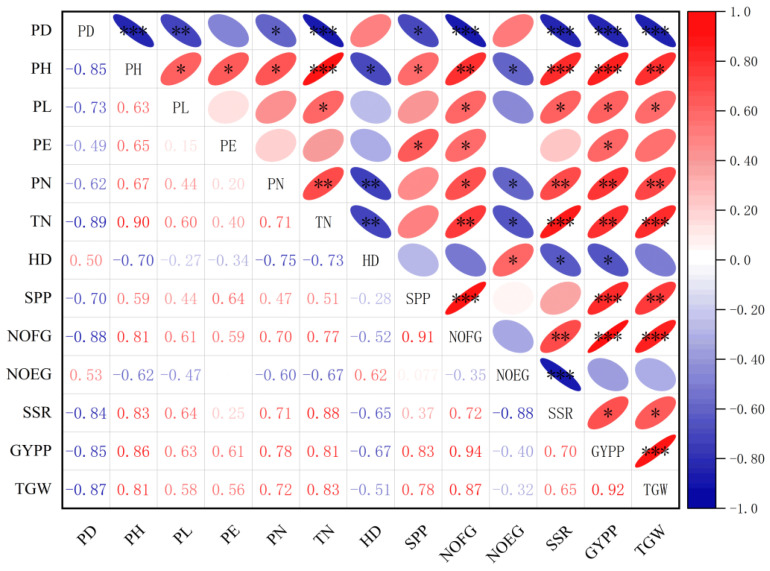
Correlation heatmap depicting the effects of different planting densities on rice growth. Blank cells indicate non-significant correlations; *, **, and *** denote significance at *p* < 0.05, *p* < 0.01, and *p* < 0.001, respectively.

Yield-related traits also displayed density-dependent reductions (Table 1): grain yield per plant and 1000-grain weight both decreased progressively with increasing planting density. Notably, despite PD-100 producing the lowest number of grains per plant (59.8 grains plant^−1^), it achieved an estimated grain yield of 304.23 g·m^−2^, which was higher than PD-30 and PD-50 but significantly lower than PD-25 (*p* < 0.01; Figure 4).

Collectively, these results indicate that planting density significantly affects rice morphological development, growth duration, and yield formation under the LED-SB platform, with high densities (PD-50 and PD-100) generally exerting inhibitory effects on most agronomic traits except growth duration.

Planting density substantially altered rice plant architecture and yield components (Table 1). As density increased from PD-25 to PD-100, all agronomic traits except heading time decreased monotonically, showing a clear threshold effect. Panicle length and filled grain number remained stable until PD-30 then decreased sharply at PD-50. Higher planting densities consistently suppressed rice growth and reduced yield. PD-30 exhibited the longest heading time (81.44 ± 1.33 days), a 15.2% significant increase relative to PD-25 (*p* < 0.05). Although phenotypic values were slightly lower in PD-30 than PD-25, ANOVA indicated no significant differences between these densities. However, beyond the critical threshold of 30 plants/tray, growth and yield were significantly suppressed. PD-100 demonstrated the feasibility of high-density propagation, maintaining 59.80 ± 12.88 grains per plant and a 64 ± 11% seed-setting rate.

Planting density showed significant negative correlations with most rice growth and yield traits under LED-SB conditions, except heading date (HD) and number of empty grains (NOEG) (Figure 4). Strong negative correlations were observed between planting density and plant height (PH, r = −0.85), tiller number (TN, r = −0.89), filled grain number (NOFG, r = −0.88), seed-setting rate (SSR, r = −0.84), grain yield per plant (GYPP, r = −0.85), and 1000-grain weight (TGW, r = −0.87) (all *p* < 0.01 or *p* < 0.001). Panicle length (PL, r = −0.73), spikelets per panicle (SPP, r = −0.70), and panicle number (PN, r = −0.62) also showed significant negative correlations with density. Heading date (HD) showed a significant positive correlation with density (r = 0.50), indicating delayed heading under high-density conditions. Similarly, empty grain number (NOEG) positively correlated with density (r = 0.53), suggesting that high density promotes the formation of unfilled grain. Increasing planting density suppressed most agronomic and yield traits, particularly tiller formation, grain filling, and grain yield. These results highlight the need for integrated management strategies to mitigate inter-plant competition in high-density systems.

### 2.3. Variation in Growth Duration of Rice Seedlings at Different Ages Following Short-Day Induction in the LED-SB Platform

**Figure 5:**
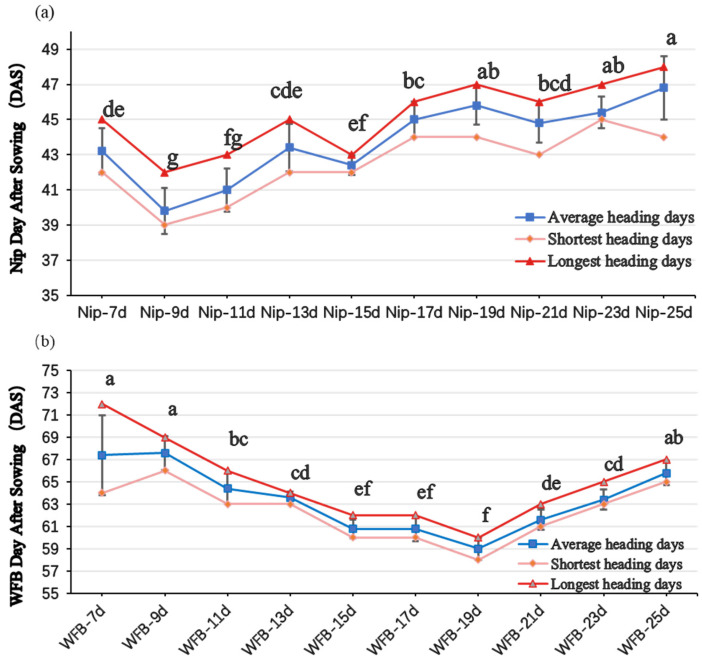
Effects of seedling age at transplantation on heading time in rice under LED short-day (LED-SB) conditions. Seedlings of (**a**) japonica cultivar ‘Nip’ and (**b**) indica cultivar ‘WFB’ were cultivated under natural light for varying durations (7–25 days) before transplantation. Plants were then subjected to 10 h short-day induction in the plant factory. Heading time was recorded as days after transplantation. Shared letters above bars indicate no significant differences (*p* > 0.05); different letters denote statistically significant differences (*p* < 0.05).

**Figure 6:**
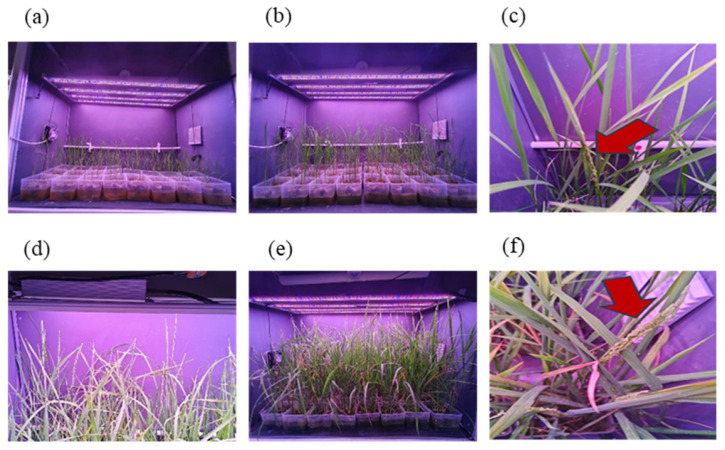
Growth dynamics of Nip and WFB under the speed breeding system. (**a**) Transplantation of rice seedlings at different ages for short-day induction; (**b**) one week after transplantation of seedlings with different ages in the plant factory; (**c**) early heading of Nip at 39 DAS; (**d**) complete heading of all Nip plants by 46 DAS; (**e**) WFB not heading yet at 46 DAS; (**f**) heading of WFB at 58 DAS. Note: The red arrows indicate the young panicles of rice plants.

The japonica cultivar Nip showed the earliest heading time (39.8 days) with the seedlings exposed to short-day induction at the age of 9 days after sowing, representing 7.9% and 15.0% reductions compared to 7-day and 25-day transplants, respectively. The indica cultivar WFB attained the earliest heading date (59.0 ± 1.0 days) with seedlings exposed to short-day induction at the age of 19 days after sowing, reducing heading time by 12.5% and 10.3% versus 7-day and 25-day seedlings, respectively (Figure 5). Relative to outdoor cultivation (100 days for Nip, 125 days for WFB), short-day induction in the plant factory at 9 DAS for Nip and 19 DAS for WFB reduced the total life cycle by 46.2% and 41.6%, respectively, enabling the fastest generation advancement. LED-SB treatment accelerated the vegetative-to-reproductive transition in Nip, with stage-specific progression detailed in Figure 6.

### 2.4. GA_3_ Treatment Promotes Germination of Early-Harvested Seeds

**Figure 7:**
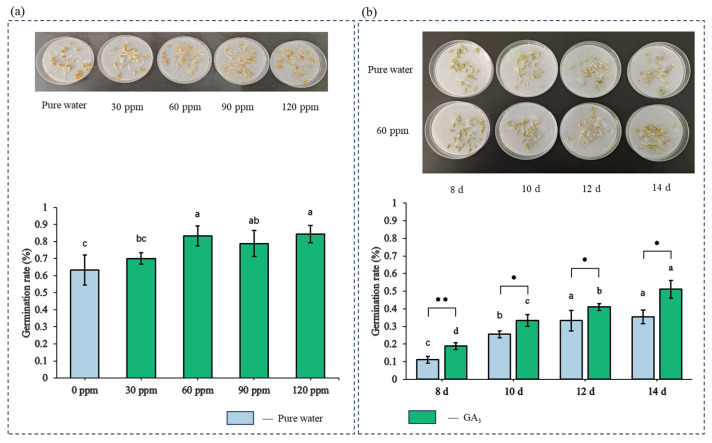
Effect of GA_3_ treatment on the germination of early-harvested rice seeds. (**a**) Concentration–response test of GA_3_ solution on germination rate using mature Nip seeds; (**b**) seeds harvested at 8, 10, 12, and 14 days after heading (DAH) were dried, screened, and grouped for treatment with purified water or 60 mg/L GA_3_ solution. Each treatment consisted of 30 seeds with three replicates. NS indicates no significant difference; *, and ** denote significance at *p* < 0.05, and *p* < 0.01, respectively; shared letters indicate no significant difference, while different letters denote significant differences.

Exogenous gibberellin (GA_3_) significantly improved germination of immature rice seeds (Figure 7). Using Nip, germination was tested for immature seeds harvested at 8, 10, 12, and 14 days after heading (DAH) and for mature seeds. Mature seeds exposed to GA_3_ gradients (0–120 mg·L^−1^) showed germination rates of 63 ± 8% to 84 ± 4%, peaking at 60 mg·L^−1^. No significant improvement occurred above 90 mg·L^−1^ (*p* > 0.05). Untreated immature seeds showed increasing germination with maturation. GA_3_ treatment (60 mg·L^−1^) increased germination rates across 8–14 DAH harvests (19 ± 2% to 51 ± 5%) versus controls (11 ± 2% to 35 ± 4%) (*p* < 0.05). Notably, 14 DAH seeds treated with 60 mg·L^−1^ GA_3_ exceeded 50% germination, a 31% increase over controls.

## 3. Discussion

Light is the primary environmental factor regulating energy acquisition and growth in rice, critically influencing yield and quality through modulation of metabolism, morphogenesis, and photosynthesis [18]. Light spectrum and intensity directly regulate photosynthetic efficiency and morpho-physiological traits, whereas photoperiod response determines heading and flowering time in short-day plants [19]. Thus, precise integration of spectrum, intensity, and photoperiod—forming the core light recipe—enables accurate growth cycle control in plant factories. Optimization of non-light factors (water, nutrition, CO_2_, hormones, substrate, density) further enhances light regulation effects, establishing a comprehensive system for industrial-scale crop propagation.

### 3.1. Light Intensity Significantly Regulates Morphogenesis Across Rice Growth Stages

Rice plants in the LED-SB platform showed superior performance in both vegetative and reproductive growth compared to natural conditions. Differential light intensity effects across growth phases reflect the stable light signaling and efficient energy supply of LED sources. Photosynthetic rate depends on both steady-state capacity and response speed to environmental fluctuations [20]. Thus, stable light conditions promote organ development and accelerate vegetative-to-reproductive transition. During BVP under continuous light (24 h/d), plant height showed no significant difference between 450 and 900 μmol·m^−2^·s^−1^ for either cultivar. The 450 μmol·m^−2^·s^−1^ treatment enhanced leaf length and width, indicating advantages for seedling vegetative development. Under PSP short-day conditions (10 h/d), plant height was greater at 450 than 900 μmol·m^−2^·s^−1^, though leaf dimensions were unaffected. This light intensity also enhanced root length and tiller number. Neither light intensity affected total growth duration (Table 2), but heading occurred earlier under 900 PPFD conditions. Plant height during the PSP was significantly greater under 450 PPFD, with leaf length also being longer. Furthermore, under 900 PPFD conditions, we observed leaf tip necrosis and marginal scorching in rice leaves. This suppression may be caused by direct photodamage at leaf–light interfaces. This multidimensional stress reduces photosynthetic carbon assimilation and disrupts resource allocation to grain filling. Dwarf germplasms optimize light energy distribution, improve light use efficiency, and show better adaptability to plant factories. Specifically, 450 μmol·m^−2^·s^−1^ promoted above-ground architecture, while 900 μmol·m^−2^·s^−1^ enhanced root elongation, tillering, and panicle development, while growth cycle shortening coincided with general agronomic trait suppression.

### 3.2. Rice Seedlings Exhibit Significant Variation in Photoperiod Sensitivity Onset Across Different Ages

Compared to 25 DAS, transplanting at 15 DAS significantly shortened heading time and total growth duration, consistent with known photoperiod acceleration in short-day crops [2]. During short-day induced floral transition, heading time showed threshold responses to seedling age. Peak photoperiod sensitivity occurred at specific stages: Nip headed earliest (39 days) with 9-day-old seedlings, while WFB headed at 58 days with 19-day-old seedlings. This contrast reflects evolutionary divergence in photoperiod regulatory networks during japonica–indica differentiation. Even at 25 DAS, both cultivars headed earlier under artificial lighting than in natural conditions, suggesting that optimized light regimes can partially compensate for genetic differences to promote flowering.

### 3.3. Seedling Tray Planting Density Modulates Rice Growth and Development in the LED-SB Platform

Conventional field planting densities are unsuitable for the spatially constrained environments of plant factories. Population size critically determines breeding efficiency in rapid breeding systems, necessitating density optimization under plant factory constraints to enable efficient speed breeding. At PD-50 and PD-100, plant height, panicle length, and tiller number were significantly suppressed. In natural canopies, shade avoidance syndrome (SAS) promotes elongation toward high-light regions to maximize light capture [21]. In plant factories, optimized vertical light distribution can mitigate shade stress and maintain growth under high densities. The plant height reduction under high density agrees with Sandhu et al. [16]. Panicle exsertion decreased dramatically with density: 45.1% at PD-50 and 88.1% at PD-100, indicating preferential inhibition of reproductive organ expansion under density stress. Although grain yield per plant declined linearly with density, yield per unit area increased due to density compensation, consistent with high-density wheat and barley systems [22]. In high-density maize, elevated density increases chlorophyllase activity and accelerates senescence, while nitrogen application reduces chlorophyll degradation, delays senescence, and promotes yield [23]. This mechanism informs coordinated light–nutrition regulation for rice under high light intensities in plant factories.

In practical breeding applications, planting density should be strategically selected according to the breeding stage. We recommend PD-100 or PD-50 for early generations (e.g., F_2_–F_4_). At this stage, the primary objective is to screen large populations for major traits such as disease resistance and basic plant architecture, where population size takes precedence despite potential delays in heading and reduced individual plant vigor. Conversely, PD-25 is recommended for advanced generations (e.g., F_5_ and beyond). When evaluating fixed or nearly fixed lines, the focus shifts to precise phenotyping for yield components and quality traits. Under these conditions, the superior per-plant performance and shorter growth cycle achieved with PD-25 become critical for accurate selection.

### 3.4. Integration of Light, Planting Density, and Phytohormone Regulation Significantly Shortens the Total Growth Duration in Rice

Regulation of sowing, LED lighting, and early post-heading harvest substantially shortened the rice growth cycle. Seedlings nursery-cultured under natural light for 9 days (Nip) or 19 days (WFB) and transferred to plant factory short-day conditions reached heading in 39.8 and 59 days, respectively. In Table 2, under 24 h LED lighting for 15 days before transplantation, heading occurred at 50.8 days (Nip) and 55.2 days (WFB). Relative to natural conditions (65.73 days for Nip, 78.2 days for WFB), natural light nursery shortened heading by 39.45% (Nip) and 29.41% (WFB). Combined with early harvest, total growth duration decreased by 46.64% (Nip) and 38.87% (WFB), demonstrating accelerated generational turnover. Although early harvesting and GA_3_ treatment shortened the cycle, germination rates remained suboptimal. Delaying harvest or extending post-heading photoperiod may improve grain maturation. The optimal GA_3_ concentration (60 mg·L^−1^) identified in this study was validated using Nip. It should be noted that phytohormone responses often vary across genotypes. Therefore, while effective for Nip, this specific GA_3_ treatment may require empirical optimization for other rice varieties, including WFB.

### 3.5. Integration of LED-SB Technology with Molecular Breeding: A Framework for Accelerated Rice Improvement

**Figure 8:**
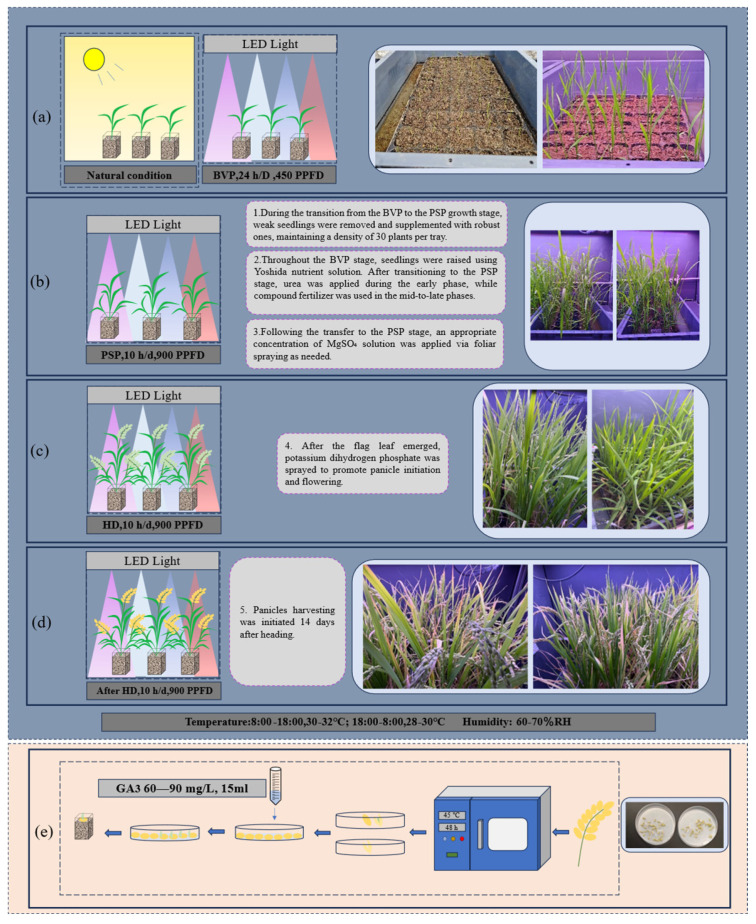
Schematic diagram of the rapid generational cycling protocol for rice. (**a**) Seedlings in the photoperiod-insensitive basic vegetative phase are nursery-grown under natural or LED light to target ages; (**b**) at the photoperiod-sensitive phase, seedlings are transplanted to the plant factory for short-day induced floral transition; (**c**) under controlled plant factory conditions, plants undergo rapid growth to heading stage; (**d**) panicles harvested at 14 days after heading; (**e**) harvested panicles dried (45 °C, 48 h), then selected per Section 4.4 (experiment 4) Seeds with intact morphology and normal embryos are retained, treated with 60 mg/L GA_3_ (15 mL, 28 °C, 75% RH, 48 h), and germinated seeds are sown in substrate to start the next cycle.

Several mature rice speed breeding (SB) protocols have been developed recently, including those by Liu, Kabade and Sandhu [3,15,16]. Our study establishes an LED plant factory platform for rice SB (Figure 8), aiming to develop a tailored LED-SB scheme for breeding accelerators. We used low-cost seedling trays and determined maximum planting density with 25 trays. A staged light-intensity strategy was applied: nursery under natural light or 450 μmol·m^−2^·s^−1^ (continuous illumination) for vegetative growth, then transfer to 900 μmol·m^−2^·s^−1^ (short day) for reproductive transition. Optimal seedling ages for short-day induction were systematically examined across rice varieties with different maturity. Nursery under natural light in South China with subsequent transplantation to LED-SB significantly shortened the growth cycle Compared to long-day crops (e.g., wheat, rapeseed), short-day crops require stricter photoperiod control. This approach (BVP nursery, PSP short-day induction, early harvest) has been applied in other short-day crops: soybean [24,25], cotton [26], and cannabis [27]. Rice growth cycle reduction was similar with natural or LED nursery light, indicating short-day induction primarily drives early heading under consistent spectrum. However, efficient rapid breeding requires precise integration of photoperiod, spectrum, and intensity into a coherent light recipe, synchronized with tailored agronomic strategies. Speed breeding has been applied in practical breeding programs, for example, Rana et al. used biotron-based SB with SNP marker-assisted selection to introgress hst1 from ‘Kaijin’ into ‘Yukinko-mai’, developing salt-tolerant ‘YNU31-2-4’ within 17 months [14]. However, high cost limits widespread adoption in rice breeding, therefore developing low-cost LED-SB platforms aligns better with breeder expectations. Using economical methods, this study demonstrates feasible rapid propagation via LED-SB, showing potential for field implementation

Although this study established a reliable LED-SB protocol, limitations remain. This work focused on LED light effects on macroscopic traits, without exploring underlying mechanisms through gene expression analysis. Furthermore, this study established a foundational speed breeding framework under controlled plant factory conditions. While it demonstrates significant potential for accelerating rice generation cycles, its broader application requires further validation across diverse rice genotypes, different facility configurations, and various breeding objectives.

## 4. Materials and Methods

### 4.1. Plant Materials

*Japonica* rice cultivar Nipponbare (Nip), *indica* rice cultivar Wufeng B (WFB) and R998 were used in this study. WFB and Nip served as the experimental materials for Experiments 1, 3, and 4, while R998 was employed for Experiment 2. All plant materials were provided by the Rice Research Institute, Guangdong Academy of Agricultural Sciences.

### 4.2. Seed Treatment and Transplantation

Selected seeds were gently agitated in sterile purified water and allowed to settle before supernatant removal. Damaged, infested, deformed, or moldy grains were manually discarded, and the remaining intact seeds were placed into mesh bags. Seeds were surface-sterilized in 3% (*v*/*v*) hydrogen peroxide for 30 min and rinsed 2–3 times with sterile purified water. Seeds were transferred to Petri dishes with 15 mL purified water and germinated in darkness (28 °C, 75% RH, 48 h). Germinated seeds were sown according to the experimental design: three seeds per planting hole were lightly embedded in autoclaved growth substrate (121 °C, 30 min). At 7 days after sowing (DAS), uniformly developed seedlings were manually thinned to select homogeneous individuals. The growth substrate was a standardized mixture of peat, vermiculite, and yellow loam (commercial formula), which was applied consistently across all treatments. Two planting configurations were adopted: (1) Configuration A: 50-cell seedling trays (540 × 280 × 45 mm), used in Experiments 1, 2, and 4; (2) Configuration B: Cultivation pots (150 × 150 × 115 mm), each containing five independent planting boxes (45 × 45 × 80 mm). This (2) configuration was exclusively used in Experiment 3 to enable independent management of seedlings with different ages.

### 4.3. Cultivation Management

A Yoshida-based dry powder nutrient solution was used as the basal nutrient supply throughout the experiment. During the seedling stage, the nutrient solution concentration was maintained at 2 g·L^−1^, with a reduced concentration of 1 g·L^−1^ during the first week after sowing; the solution was replaced every week. Starting from 15 days after sowing (DAS), 2 g·L^−1^ urea was supplemented weekly as a topdressing. At the jointing stage, weekly application of 2 g·L^−1^ compound fertilizer was initiated. Prior to the emergence of the flag leaf tip, a 1:1 (*w*/*w*) mixture of 2 g·L^−1^ KH_2_PO_4_ and 2 g·L^−1^ urea was applied weekly. Following the full expansion off lag leaf, 2 g·L^−1^ KH_2_PO_4_ was foliar-sprayed weekly until the heading stage. Based on in situ observations of plant growth performance, micronutrient supplementation and dynamic adjustments of nutrient concentrations were conducted to ensure precise nutrient supply matching plant developmental demands.

### 4.4. Experimental Treatments

Experiment 1. Plants were grown under controlled conditions: 32–30 °C/30–28 °C day/night temperature, 60–70% relative humidity (RH), and three light intensity levels (natural light, 450 μmol·m^−2^·s^−1^, 900 μmol·m^−2^·s^−1^) applied during both BVP (24 h/d) and PSP (10 h/d). Each light intensity treatment was assigned to a separate growth chamber. Uniformly germinated seeds of rice cultivars ‘Nip’ and ‘WFB’ were transplanted (50 plants each). Plant height, root length, leaf length, leaf width, and tiller number were measured at 15 and 45 days after sowing (DAS), with five technical replicates per trait, using a completely randomized design.

Experiment 2. Plants were grown in a plant factory under uniform controlled conditions: 10 h light/14 h dark photoperiod, 900 μmol·m^−2^·s^−1^ photosynthetic photon flux density (PPFD), 32–30 °C/30–28 °C (day/night) temperature, and 60–70% relative humidity (RH). Four planting densities were tested: PD-25 (25 plants/tray), PD-30 (30 plants/tray), PD-50 (50 plants/tray), and PD-100 (100 plants/tray), each assigned to an independent growth chamber. All treatments used standardized 50-cell seedling trays (540 × 280 × 45 mm) (Figure 4). At harvest, the following parameters were determined with five technical replicates each: plant height, panicle length, panicle exsertion, panicle number, tiller number, heading date, spikelets per panicle, filled/unfilled grain numbers, seed-setting rate, yield per plant, and 1000-grain weight, using a completely randomized design.

Experiment 3. Plants were grown in a plant factory under controlled environmental conditions: photoperiod of 10 h light/14 h dark, photosynthetic photon flux density (PPFD) of 900 μmol·m^−2^·s^−1^, day/night temperatures of 32–30 °C/30–28 °C, and relative humidity (RH) of 60–70%. The effects of transplanting were systematically evaluated using the cultivars japonica ‘Nip’ and indica ‘WFB’. Ten seedling age gradients (7–25 days) were set for the experiment. After germination, seedlings were nursery-grown under natural light until reaching the target ages, and then transferred to the plant factory for short-day induction. Seedlings were cultivated in pots, each containing five independent planting boxes (one plant per box), resulting in five biological replicates per seedling age. Heading dates of all plants were recorded. Five plants for each treatment were placed in three replications using a completely randomized design.

Experiment 4. Intact panicles were harvested at 8, 10, 12, and 14 days after heading (DAH) and dried at 45 °C for 48 h. Empty grains (cracked hulls or non-embryonic seeds) were discarded, and seeds with intact hulls or visible embryos were retained. The dried seeds were subjected to two treatments: purified water (control) and 60 mg·L^−1^ GA_3_. For each treatment, seeds were mixed with 15 mL of the respective solution. A separate germination assay was conducted using mature ‘Nip’ seeds, with GA_3_ concentration gradients of 0, 30, 60, 90, and 120 mg·L^−1^. Using a completely randomized design, each treatment was replicated three times with 30 seeds per replication.

### 4.5. Light Environmental Conditions

**Figure 9:**
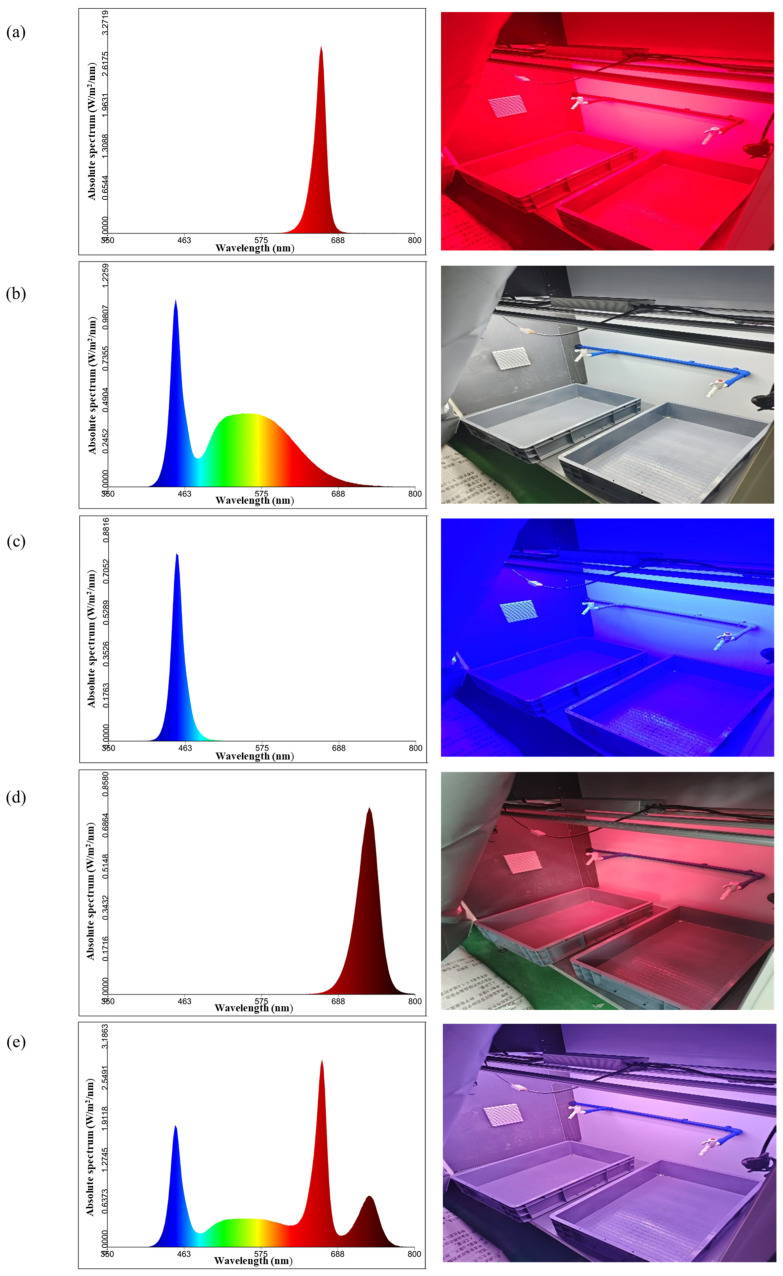
Spectral intensity of different LED wavebands measured at 50 cm below the light source. (**a**) Red LED: PPFD = 401 μmol·m^−2^·s^−1^; (**b**) White LED: PPFD = 431 μmol·m^−2^·s^−1^; (**c**) Blue LED: PPFD = 77 μmol·m^−2^·s^−1^; (**d**) Far-red LED: PPFD = 13 μmol·m^−2^·s^−1^; (**e**) Full-spectrum LED: PPFD = 917 μmol·m^−2^·s^−1^.

The plant factory system comprised 16 independent growth chambers of identical dimensions. Each chamber was equipped with an adjustable overhead LED lighting module, integrating four spectral channels: red (660 nm), blue (450 nm), white (full spectrum), and far-red (730 nm). A central control system regulated the spectral ratio, photosynthetic photon flux density (PPFD), and photoperiod for each channel in real time. Each LED module was independently connected to a time-controlled switch, enabling microsecond-precision light control (Figure 9).

### 4.6. Data Analysis

Data were analyzed using Excel 2019, SPSS 27.0, and Origin 2024. Normality of data and homogeneity of variance were verified via the Shapiro–Wilk test and Levene’s test, respectively. One-way analysis of variance (ANOVA) with a significance level of α = 0.05 was used to evaluate differences among groups. Significant differences were further separated using Duncan’s multiple range test. Pearson’s correlation analysis was conducted in Origin 2024. All data are presented as mean ± standard deviation (SD).

## 5. Conclusions

Light environment regulation is the primary factor shortening the rice growth cycle in plant factories. Reasonable planting density coordinates the growth period and plant population, while early harvesting compresses the seed-to-seed cycle. Coupled optimization of light, density, and hormone-mediated harvest synergistically accelerates growth. Using a multi-factor control platform, this study integrated light environment, planting density, and exogenous GA_3_ to establish a rapid rice propagation system. Our key findings include the following: (1) gradient light intensity (450 PPFD in BVP, 900 PPFD in PSP) balances vegetative growth and floral transition; (2) high-density planting (25 plants/tray) shortens total growth duration; (3) GA_3_ treatment (60 ppm) increases immature seed germination to over 50%, alleviating endosperm development constraints. The integrated “light–density–hormone” strategy reduced the generation time to 54 days for the japonica Nip and to 70 days for the indica WFB. The protocol used was as follows: During BVP, either nursery cultivation under natural light or continuous illumination (24 h/d, 450 PPFD) in a plant factory was applied. During PSP, short-day conditions (10 h/d, 900 PPFD) were applied to induce flowering. In conclusion, this study established a reliable rapid breeding protocol for rice, providing essential data and technical support for scalable applications and intelligent environmental optimization in plant factories, and shows significant potential for implementation in rice breeding programs to accelerate varietal improvement.

## Figures and Tables

**Table 1:** Effects of different planting densities on rice growth and yield-related traits under the LED-SB platform.

Traits	Planting Density (PD)				*p*-Value
	PD-25	PD-30	PD-50	PD-100	
Plant height (cm, PH)	75.16 ± 1.48 a	66.24 ± 3.46 b	62.48 ± 3.11 c	61.16 ± 3.73 c	***
Panicle length (cm, PL)	21.87 ± 0.59 a	20.88 ± 1.96 ab	20.42 ± 1.24 b	18.50 ± 1.82 c	***
Panicle exsertion (cm, PE)	3.55 ± 2.42 a	1.95 ± 2.34 ab	−2.16 ± 1.96 b	0.42 ± 1.17 c	***
Panicle number (PN)	3.4 ± 0.97 a	1.9 ± 0.74 b	1.7 ± 0.48 b	1.00±0 c	***
Tiller number (TN)	5.30 ± 0.95 a	3.70 ± 0.82 b	3.20 ± 0.79 b	1.60 ± 0.70 c	***
Heading date (HD)	70.70 ± 2.58 c	81.44 ± 1.33 a	77.89 ± 1.76 b	81.30 ± 1.89 a	***
Spikelets per panicle (SPP)	145.80 ± 21.07 a	128.67 ± 15.13 a	99.11 ± 14.02 b	95.40 ± 21.18 b	***
Number of filled grains (NOFG)	129.10 ± 21.62 a	109.67 ± 16.05 b	74.67 ± 11.02 c	59.80 ± 12.88 c	***
Number of empty grains (NOEG)	16.70 ± 7.32 b	19.00 ± 10.64 b	24.44 ± 13.99 ab	35.60 ± 16.80 a	*
Seed setting rate (%, SSR)	0.88 ± 0.05 a	0.85 ± 0.08 a	0.76 ± 0.11 b	0.64 ± 0.11 c	***
Grain yield per plant (g, GYPP)	2.22 ± 0.36 a	1.48 ± 0.22 b	0.82 ± 0.20 c	0.46 ± 0.12 d	***
Thousand-grain weight (g, TGW)	21.18 ± 0.93 a	20.62 ± 0.25 a	18.00 ± 0.44 b	17.06 ± 0.65 b	***

Note: Comparisons within the same row were conducted using Duncan’s multiple range test. Different letters indicate significant differences, while the same letter denotes no significant difference; * and *** denote significance at *p* < 0.05, and *p* < 0.001, respectively.

**Table 2:** Growth duration of Nip and WFB under the LED-SB platform.

Group	SG	Vegetative Phase		Heading Days	One Generation in Field and LED-SB
		BVP	PSP		
CK-Nip	2	Natural Light		65.73 ± 1.03 a	100.73
450-Nip	2	15 (450 PPFD)	15 DAS–HD (900 PPFD)	51.40 ± 0.74 b	65.4
450–900-Nip	2	15 (450 PPFD)	15 DAS–HD (900 PPFD)	50.80 ± 0.68 b	64.8
CK-WFB	2	Natural Light		78.20 ± 1.26 a	113.2
450-WFB	2	15 (450 PPFD)	15 DAS–HD (900 PPFD)	55.53 ± 0.83 b	69.53
450–900-WFB	2	15 (450 PPFD)	15 DAS–HD (900 PPFD)	55.20 ± 0.94 b	69.2
NL-900-Nip	2	9-NL	9 DAS–HD (900 PPFD)	39.8 ± 1.30	53.8

Abbreviations: SG, soaking and germination; NL, natural light; DAS, days after sowing; HD, heading days. Note: Shared letters above bars indicate no significant differences (*p* ≥ 0.05); different letters denote statistically significant differences (*p* < 0.05).

## Data Availability

All data generated or analyzed during this study are included in this published article.

## References

[1] Hickey L.T., Hafeez A.N., Robinson H., Jackson S.A., Leal-Bertioli S.C.M., Tester M., Gao C., Godwin I.D., Hayes B.J., Wulff B.B.H. (2019). breeding crops to feed 10 billion. Nat. Biotechnol..

[2] Watson A., Ghosh S., Williams M.J., Cuddy W.S., Simmonds J., Rey M.-D., Asyraf Md Hatta M., Hinchliffe A., Steed A., Reynolds D. (2018). Speed breeding is a powerful tool to accelerate crop research and breeding. Nat. Plants.

[3] Liu Y., Li Z.-G., Cheng H., Yang X., Li M.-Y., Liu H.-Y., Gan R.-Y., Yang Q.-C. (2025). Plant factory speed breeding significantly shortens rice generation time and enhances metabolic diversity. Engineering.

[4] Dsouza A., Newman L., Graham T., Fraser E.D.G. (2023). Exploring the landscape of controlled environment agriculture research: A systematic scoping review of trends and topics. Agric. Syst..

[5] Jang I.T., Lee J.H., Shin E.J., Nam S.Y. (2023). Evaluation of growth, flowering, and chlorophyll fluorescence responses of *Viola cornuta* cv. penny red wing according to spectral power distributions. J. People Plants Environ..

[6] Miao C., Yang S.J., Xu J., Wang H., Zhang Y.X., Cui J.W., Zhang H.M., Jin H.J., Lu P.L., He L.Z. (2023). Effects of light intensity on growth and quality of lettuce and spinach cultivars in a plant factory. Plants.

[7] Kang J.H., KrishnaKumar S., Atulba S.L.S., Jeong B.R., Hwang S.J. (2013). Light intensity and photoperiod influence the growth and development of hydroponically grown leaf lettuce in a closed-type plant factory system. Hortic. Environ. Biotechnol..

[8] Thongtip A., Mosaleeyanon K., Korinsak S., Toojinda T., Darwell C.T., Chutimanukul P., Chutimanukul P. (2022). Promotion of seed germination and early plant growth by KNO_3_ and light spectra in *Ocimum tenuiflorum* using a plant factory. Sci. Rep..

[9] Zheng J.F., Ji F., He D.X., Niu G.H. (2019). Effect of light intensity on rooting and growth of hydroponic strawberry runner plants in a led plant factory. Agronomy.

[10] He R., Ju J., Liu K.Z., Song J.L., Zhang S.C., Zhang M.G., Hu Y.Z., Liu X.J., Li Y.M., Liu H.C. (2024). Technology of plant factory for vegetable crop speed breeding. Front. Plant Sci..

[11] Song Y., Duan X., Wang P., Li X., Yuan X., Wang Z., Wan L., Yang G., Hong D. (2021). Comprehensive speed breeding: A high-throughput and rapid generation system for long-day crops. Plant Biotechnol. J..

[12] Cha J.-K., Park H., Choi C., Kwon Y., Lee S.-M., Oh K.-W., Ko J.-M., Kwon S.-W., Lee J.-H. (2023). Acceleration of wheat breeding: Enhancing efficiency and practical application of the speed breeding system. Plant Methods.

[13] Schilling S., Melzer R., Dowling C.A., Shi J., Muldoon S., McCabe P.F. (2022). A protocol for rapid generation cycling (speed breeding) of hemp (*Cannabis sativa*) for research and agriculture. Plant J..

[14] Rana M.M., Takamatsu T., Baslam M., Kaneko K., Itoh K., Harada N., Sugiyama T., Ohnishi T., Kinoshita T., Takagi H. (2019). Salt tolerance improvement in rice through efficient SNP marker-assisted selection coupled with speed-breeding. Int. J. Mol. Sci..

[15] Kabade P.G., Dixit S., Singh U.M., Alam S., Bhosale S., Kumar S., Singh S.K., Badri J., Varma N.R.G., Chetia S. (2023). SpeedFlower: A comprehensive speed breeding protocol for indica and japonica rice. Plant Biotechnol. J..

[16] Sandhu N., Singh J., Pruthi G., Verma V.K., Raigar O.P., Bains N.S., Chhuneja P., Kumar A. (2024). SpeedyPaddy: A revolutionized cost-effective protocol for large scale offseason advancement of rice germplasm. Plant Methods.

[17] Jähne F., Hahn V., Würschum T., Leiser W.L. (2020). Speed breeding short-day crops by LED-controlled light schemes. Theor. Appl. Genet..

[18] Shafiq I., Hussain S., Raza M.A., Iqbal N., Asghar M.A., Raza A., Fan Y.-f., Mumtaz M., Shoaib M., Ansar M. (2021). Crop photosynthetic response to light quality and light intensity. J. Integr. Agric..

[19] Vicentini G., Biancucci M., Mineri L., Chirivì D., Giaume F., Miao Y., Kyozuka J., Brambilla V., Betti C., Fornara F. (2023). Environmental control of rice flowering time. Plant Commun..

[20] Yamori W. (2016). Photosynthetic response to fluctuating environments and photoprotective strategies under abiotic stress. J. Plant Res..

[21] Huber M., Nieuwendijk N.M., Pantazopoulou C.K., Pierik R. (2020). Light signalling shapes plant–plant interactions in dense canopies. Plant Cell Environ..

[22] Ghosh S., Watson A., Gonzalez-Navarro O.E., Ramirez-Gonzalez R.H., Yanes L., Mendoza-Suárez M., Simmonds J., Wells R., Rayner T., Green P. (2018). Speed breeding in growth chambers and glasshouses for crop breeding and model plant research. Nat. Protoc..

[23] Lan T., Du L., Wang X., Zhan X., Liu Q., Wei G., Lyu C., Liu F., Gao J., Feng D. (2024). Synergistic effects of planting density and nitrogen fertilization on chlorophyll degradation and leaf senescence after silking in maize. Crop J..

[24] Rajendran A., Ramlal A., Raju D., Saini M., Bishnoi P., Subramaniam S. (2025). Photoperiod-mediated rapid generation advancement in soybean (*Glycine max* (L.) Merr.). Photosynth. Res..

[25] Taku M., Saini M., Kumar R., Debbarma P., Rathod N.K.K., Onteddu R., Sharma D., Pandey R., Gaikwad K., Lal S.K. (2024). Modified speed breeding approach reduced breeding cycle to less than half in vegetable soybean [*Glycine max* (L.) Merr.]. Physiol. Mol. Biol. Plants.

[26] Wang G., Sun Z., Yang J., Ma Q., Wang X., Ke H., Huang X., Zhang L., Wang G., Gu Q. (2025). The speed breeding technology of five generations per year in cotton. Theor. Appl. Genet..

[27] Pierroz G. (2023). Need for speed: A breakthrough speed breeding protocol for hemp. Plant J..

